# Treatments for kinesiophobia in people with chronic pain: A scoping review

**DOI:** 10.3389/fnbeh.2022.933483

**Published:** 2022-09-20

**Authors:** Martine Bordeleau, Matthieu Vincenot, Salomé Lefevre, Arnaud Duport, Lucas Seggio, Tomy Breton, Thierry Lelard, Eric Serra, Nathalie Roussel, Jeremy Fonseca Das Neves, Guillaume Léonard

**Affiliations:** ^1^Research Centre on Aging, CIUSSS de l’Estrie – CHUS, Sherbrooke, QC, Canada; ^2^Faculty of Medicine and Health Sciences, Université de Sherbrooke, Sherbrooke, QC, Canada; ^3^UR UPJV 3300 APERE Adaptation Physiologiques à l’Exercice et Réadaptation à l’Effort, Université de Picardie Jules Verne, Amiens, France; ^4^Institut d’Ingénierie pour la Santé, UFR de Médecine, Université de Picardie Jules Verne, Amiens, France; ^5^URePSSS – Unité de Recherche Pluridisciplinaire Sport, Santé, Société (ULR 7369), Université du Littoral Côte d’Opale, Université de Lille, Université d’Artois, Calais, France; ^6^Centre d’Etude et de Traitement de la Douleur, Center Hospitalier Universitaire Amiens-Picardie, Amiens, France; ^7^Laboratoire PSITEC EA 4072, Université de Lille, Lille, France; ^8^Department of Rehabilitation Sciences and Physiotherapy (MOVANT), Faculty of Medicine and Health Sciences, University of Antwerp, Antwerp, Belgium; ^9^Psychiatrie de Liaison, Center Hospitalier Universitaire Amiens-Picardie, Amiens, France; ^10^School of Rehabilitation, Faculty of Medicine and Health Sciences, Université de Sherbrooke, Sherbrooke, QC, Canada

**Keywords:** kinesiophobia, fear of movement, chronic pain, scoping review, randomized controlled trial

## Abstract

Kinesiophobia is associated with pain intensity in people suffering from chronic pain. The number of publications highlighting this relationship has increased significantly in recent years, emphasizing the importance of investigating and synthesizing research evidence on this topic. The purpose of this scoping review was to answer the following questions: (1) What types of interventions have been or are currently being studied in randomized controlled trials (RCTs) for the management of kinesiophobia in patients with chronic pain? (2) What chronic pain conditions are targeted by these interventions? (3) What assessment tools for kinesiophobia are used in these interventions? According to the studies reviewed, (1) physical exercise is the most commonly used approach for managing irrational fear of movement, (2) interventions for kinesiophobia have primarily focused on musculoskeletal pain conditions, particularly low back pain and neck pain, and (3) the Tampa Scale of Kinesiophobia is the most commonly used tool for measuring kinesiophobia. Future RCTs should consider multidisciplinary interventions that can help patients confront their irrational fear of movement while taking into account the patient’s personal biological, psychological, and social experiences with pain and kinesiophobia.

## Introduction

Chronic pain is a prominent cause of disability worldwide, as well as one of the most common reasons for medical visits and absenteeism from work (Vos et al., 2012; Hoy et al., 2014). Chronic pain has several cognitive, emotional, behavioral, and functional impacts that influence the clinical course and the treatment outcome (Linton and Shaw, 2011; Giusti et al., 2020; Varallo et al., 2021b). According to the fear-avoidance model, individual who experience acute pain may get trapped in a vicious cycle of chronic incapacity and suffering due to their cognitive, emotional, behavioral, and functional responses to pain (Crombez et al., 2012). This model states that when a painful event is perceived as threatening, it can lead to catastrophizing thoughts that movement and physical activity will result in further pain and injury (Larsson et al., 2016). One component of this model includes fear of movement, or kinesiophobia, “in which a patient has an excessive, irrational, and debilitating fear of physical movement and activity resulting from a feeling of vulnerability to painful injury or re-injury” (Kori et al., 1990; Vlaeyen et al., 1995). Kinesiophobia, which affects between 51 and 72% of patients with chronic pain (Lundberg et al., 2006; Bränström and Fahlström, 2008; Perrot et al., 2018), promotes hypervigilance and worsens disability, leading to increased pain sensation (Vlaeyen and Linton, 2012). In contrast to other phobias, where individuals are generally aware of the irrationality of their fear, people with kinesiophobia believe that avoiding movement is an appropriate response, resulting in deleterious behaviors and decreased overall functional ability (Lethem et al., 1983; Desrosiers, 2018; Trinderup et al., 2018). Kinesiophobia is associated with pain intensity and disability in people suffering from chronic pain (Varallo et al., 2020, 2021a). Assessing and acting on kinesiophobia may be essential considering that physical exercise is an important component of rehabilitation treatment and high levels of kinesiophobia might compromise treatment adherence.

**FIGURE 1:**
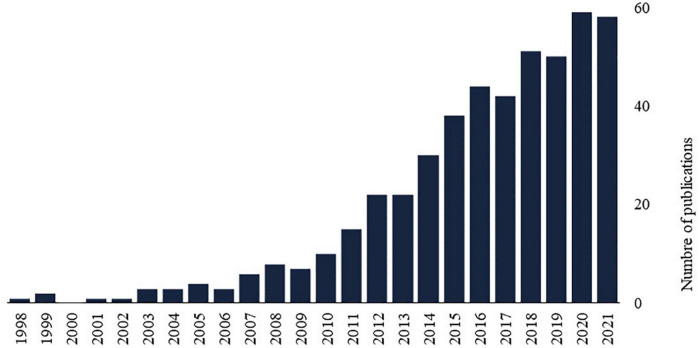
The number of publications on chronic pain and kinesiophobia by year available on PubMed (Medline) counts all publications dates for a citation as supplied by the publisher, e.g., print and electronic publication dates. Search query: (“kinesiophobia” OR “fear of movement”) AND “chronic pain”.

Recent years have witnessed a significant increase in the number of publications on the relationship between chronic pain and kinesiophobia (Figure 1), emphasizing the importance to investigate and synthesize research evidence on this topic. Up to now, five systematic reviews and meta-analyses, published between 2018 and 2021, have evaluated the effect of different interventions on kinesiophobia in patient with different pain conditions, including exercise training (Domingues de Freitas et al., 2020; Hanel et al., 2020), pain education (Tegner et al., 2018; Watson et al., 2019), and manual therapy (Kamonseki et al., 2021). All these reviews included articles that assessed fear of movement, regardless of whether kinesiophobia was considered a primary or secondary outcome. Given that previous reviews focused on specific interventions and/or chronic pain conditions, the goal of our scoping review was to map out the literature on treatments for kinesiophobia in people suffering from any type of chronic pain condition. A second goal of the review was to identify gaps in the literature as well as potential directions for future research. Our review questions were as follows:

• What types of interventions have been or are currently being studied in RCTs for the management of kinesiophobia in patients with chronic pain?
• What chronic pain conditions are targeted by these interventions?
• What assessment tools for kinesiophobia are used in these interventions?

## Materials and methods

### Design

This scoping review protocol was conducted according to the Joanna Briggs Institute Critical Appraisal tools (Peters et al., 2017) and was registered in Open Science Framework (doi: 10.17605/OSF.IO/KTJ84).^1^ This review is reported following Preferred Reporting Items for Systematic Review and Meta-Analysis (PRISMA) guidelines – extension for scoping review (Tricco et al., 2018).

### Search strategy

Pertinent studies were extracted from CINAHL, Cochrane, Scopus, Pedro, OTseeker, AMED, OTDBASE, and Medline (PubMed) between database inception and February 15, 2022. The search strategy focused on keywords related to “pain,” “kinesiophobia,” and “randomized controlled trial.” The search strategy was reviewed by an expert librarian using the Peer Review of Electronic Search Strategies (PRESS) checklist and modified as required (McGowan et al., 2016). An example of the full search strategy is presented in Supplementary Table 1 (refer Supplementary material).

### Study selection

References were gathered and duplicates were removed using EndNote (version X9, Thomson Reuters, 2019). In an initial screening, the references were separated into two groups of two independent reviewers (SL and MV, LS and AD) and eligible studies were selected based on titles and abstracts. In a second screening based on full texts, three groups of two independent co-authors (SL and MV, LS and AD, TB and MB) selected eligible studies. Discrepancies in these two selection steps were resolved by consensus between the two co-authors of the given group. A third independent author was consulted in the event of disagreement between these two co-authors (MB or GL).

### Eligibility criteria

The PCC approach (Population, Concept, and Context) was used to establish eligibility criteria, where “Population” referred to adults (>18 years old) with chronic pain (>3 months), “Concept” to any treatment for kinesiophobia, and “Context” to French or English peer-reviewed clinical articles from any country describing RCT conducted in any type of setting (e.g., laboratory, private clinic, rehabilitation center, hospital) with kinesiophobia as the primary outcome measure. When it was unclear whether the kinesiophobia measure was the primary outcome measure, an independent reviewer classified them according to their judgment. The presence of a comparator (no intervention, active/sham/placebo comparator) was required for study inclusion and randomized uncontrolled trials (i.e., studies comparing two experimental groups) were excluded. Studies evaluating the effects of postoperative interventions on kinesiophobia were also excluded. These studies were excluded due to the possibility that the operation would cause acute pain, eliciting a natural fear of movement during this stage of wound healing. Additionally, it is probable that these patients no longer experience pain following surgery and thus do not meet the criteria for patients with chronic pain.

### Data charting

Prior to data charting, the authors developed and reviewed a comprehensive data extraction tool that included descriptions of the extraction categories. The following entries were collected:

• descriptive information about the article, including the authors, publication year, aim of the study, geographic location of the study (if not listed, location of the affiliation of the first author), study design, funding source, and study registration number;
• information regarding the participants (number of participants included in the analysis, pain condition, sex);
• information on the experimental and control interventions (description, number and duration of session, duration of the intervention, follow-up);
• information on the evaluation tool used to assess kinesiophobia.

The data were charted in Microsoft Excel (Microsoft Excel, Microsoft Corporation, Washington, United States). Data charting was completed for all included studies independently by 3 groups of 2 reviewers (SL and MV, LS and AD, TB and MB, who each charted data for one-third of the studies). Data charting files were compared between reviewers and discrepancies were resolved by consensus with a third author (GL).

### Summarizing the findings

Microsoft Excel was used to calculate descriptive statistics (e.g., totals, percentages) and to create figures to summarize the data. Descriptive information on all included studies was examined together.

## Results

### Article selection

**FIGURE 2:**
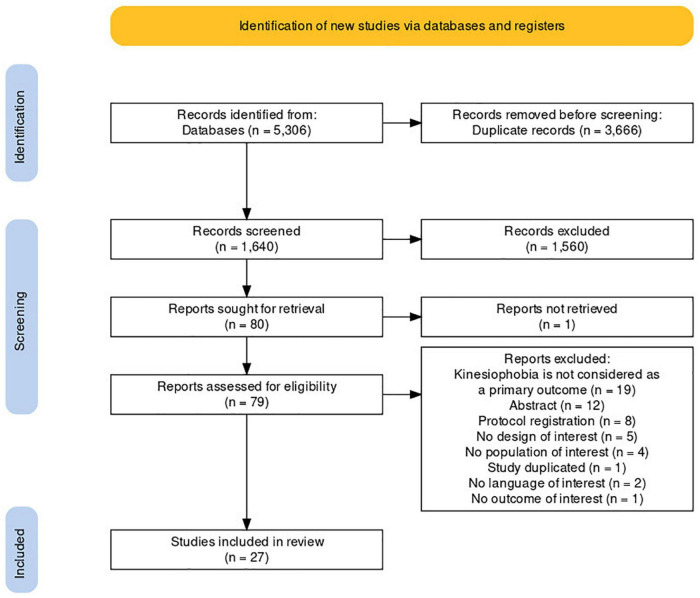
Flow diagram depicting the flow of information through the various stages of the review. This figure was created by using a customizable online tool flow diagram that adheres to PRISMA 2020 standards (Haddaway et al., 2020).

Our search strategy yielded 1,640 unique citations from which 79 articles were retrieved (Figure 2). Of these, 27 studies fulfilled the selection criteria and were included in the scoping review, while 52 were excluded (Figure 2). Our extraction and analysis data sheet is available as Supplementary material.

### Characteristics of included studies

The characteristics of the 27 peer-reviewed RCTs that have considered kinesiophobia as a primary outcome are summarized in Table 1. These articles included a total of 1,382 patients with chronic pain (759 included in experimental groups and 623 included in control groups), the majority of whom were women (67%). They were all published in English between 2006 and 2022 by research teams from Turkey (*n* = 9, 33%), Spain (*n* = 5, 19%), Iran (*n* = 3, 11%), United States (*n* = 2, 7%), and other countries (Cyprus, Egypt, the Netherlands, Nigeria, Sweden, United Kingdom). Eleven studies mentioned receiving funding from non-profit organizations (41%) and eight stated that they did not have a funding source (30%); this information was not provided for the remaining studies. Sixty percent of the included studies had registered their research protocol on open access web-based resources such as clinicaltrial.gov.

**TABLE 1:** Characteristics of the RCT included, shown in chronological order.

References	Country	Population group (*n*^†^), % male	Evaluation of kinesiophobia as an eligibility criterion?	Kinesiophobia assessment tool	Intervention	Period of the intervention	Follow-up	Study registration ID	Funding source
Kesik et al., 2022	Turkey	– EG1: multiple sclerosis (26), 23% – EG2: multiple sclerosis (27), 30% – CG: multiple sclerosis (27), 30%	No	TSK	– EG1: usual treatment and progressive muscle relaxation at home – EG2: usual treatment and Benson relaxation technique at home – CG: usual treatment and a single-time attention-matched education on living with multiple sclerosis	– EG1: 84 sessions, 60 min per session, over 12 weeks – EG2: 84 sessions, 60 min per session, over 12 weeks – CG: 12 weeks	2 weeks post intervention	NCT04833673	Non-profit
Lara-Palomo et al., 2022	Spain	– EG: cLBP (35), 23% – CG: cLBP (39), 30%	No	TSK	– EG: e-health rehabilitation program involving McKenzie exercises and TENS – CG: home rehabilitation program involving McKenzie exercises and TENS	– EG: 24 sessions, 60 min, over 8 weeks – CG: 24 sessions, 60 min, over 8 weeks	6 months post intervention*	NCT03469024	Non-profit
Akodu et al., 2021	Nigeria	– EG1: cNP (17), 41% – EG2: cNP (14), 36% – CG: cNP (14), 50%	No	TSK	– EG1: neck stabilization exercise – EG2: Pilate exercise – CG: dynamic isometric exercise	– EG1: 16 sessions, 30 min per session, over 8 weeks – EG2: 16 sessions, 30 min per session, over 8 weeks – CG: 16 session, 30 min per session, 8 weeks	None	PACTR20180 7573146508	Not reported
Bagheri et al., 2021	Iran	– EG: cPFP (15), 0% – CG: cPFP (15), 0%	No	TSK	– EG: mindfulness at home and isotonic and isometric exercises in clinic – CG: isotonic and isometric exercises in clinic	– EG: 56 sessions, 45 min per session, over 8 weeks (mindfulness) and 54 sessions, 60–90 min per session, over 18 weeks (exercises) – CG: 54 sessions, 60–90 min per session, over 18 weeks	2 months post intervention	UMIN00 0035347	Non-profit
Gül et al., 2021	Turkey	– EG: cLBP (16), NA – CG: cLBP (15), NA	No	TSK	– EG: PNE and physiotherapy (hot-pack, ultrasound, TENS, home-based exercise program) – CG: physiotherapy (hot-pack, ultrasound, TENS, home-based exercise program)	– EG: 6 sessions, 40 min per session, over 3 weeks (PNE) and 15 sessions, 90 min per session, over 3 weeks (physiotherapy) – CG: 15 sessions, 90 min per session, over 3 weeks	None	None	Non-profit
James et al., 2021	United Kingdom	– EG: cPFP (12), 17% – CG: cPFP (12), 33%	No	TSK	– EG: pain education and physiotherapy – CG: physiotherapy	– EG: 30 min (pain education) and 12 weeks (physiotherapy) – CG: 12 weeks	None	NCT03784339	Not reported
Moraes et al., 2021	Brazil	– EG1: cLBP (27), 48% – EG2: cLBP (27), 52% – CG: cLBP (27), 48%	Yes, inclusion criteria (TSK ≥ 51 points to be included)	TSK	– EG1: pain education, pain exposure and usual treatment (medical consultation and pharmacological treatment) – EG2: pain education and usual treatment – CG: usual treatment	– EG1: 3 sessions, over 3 weeks (education) and 3 sessions, over 3 weeks (exposure) – EG2: 3 sessions, over 3 weeks – CG: 3 weeks	None	None	Not reported
Nambi et al., 2021	Egypt	– EG1: cLBP (18), 100% – EG2: cLBP (18), 100% – CG: cLBP (18), 100%	No	TSK	– EG1: balance training through VR and exercises at home – EG2: balance training through a Swiss ball and exercises at home – CG: conventional balance training and exercises at home	– EG1: 20 sessions, 30 min per session, over 4 weeks – EG2: 20 sessions, over 4 weeks – CG: EG2: 20 sessions, over 4 weeks	6 months post intervention	None	Non-profit
Javdaneh et al., 2021	Iran	– EG1: cNP (24), 54% – EG2: cNP (24), 50% – CG: cNP (24), 46%	No	TSK	– EG1: neck stabilization exercise – EG2: motor imagery – CG: no intervention	– EG1: 18 sessions, 40–50 min per session, over 6 weeks – EG2: 18 sessions, 25 min per session, over 6 weeks – CG: over 6 weeks	None	None	Not reported
Reynolds et al., 2020	United States	– EG: TMD (25), 20% – CG: TMD (25), 8%	No	TSK-TMD	– EG: cervical manipulation with thrust, behavioral education, soft tissue mobilization and home exercises – CG: cervical manipulation without thrust, behavioral education, soft tissue mobilization and home exercises	– EG: 16 sessions, over 4 weeks – CG: 16 sessions, over 4 weeks	None	NCT03300297	Non-profit
Aydoğan Arslan et al., 2020	Turkey	– EG: cKP (21), 52% – CG: cKP (17), 35%	No	TSK	– EG: NMES and physiotherapy (hot pack, ultrasound, TENS, exercises program) – CG: physiotherapy (hot pack, ultrasound, TENS, exercises program)	– EG: 10 sessions, over 2 weeks – CG: 10 sessions, over 2 weeks	None	None	None
Gulsen et al., 2020	Turkey	– EG: fibromyalgia (8), 0% – CG: fibromyalgia (8), 0%	No	TSK	– EG: balance and mobility training through VR training and physiotherapy (aerobic and Pilates training) – CG: physiotherapy (aerobic and Pilates training)	– EG: 16 sessions, 80 min, overs 8 weeks – CG: 16 sessions, 80 min, overs 8 weeks	None	None	Non-profit
	Iran	– EG: cLBP (73), NIA – CG: cLBP (74), NIA	No	TSK	– EG: dry needling and intramuscular electrical stimulation with kinesiology tapping – CG: dry needling and intramuscular electrical stimulation	– EG: 12 sessions, 1 h, overs 4 weeks – CG: 12 sessions, 1 h, overs 4 weeks	None	IRCT20140616 9440N4	Non-profit**
Özer and Toprak Çelenay, 2019	Turkey	– EG: cNP (28), 17% – CG: cNP (30), 21%	No	TSK	– EG: neck stabilization exercises and progressive muscle relaxation – CG: neck stabilization exercises	– EG: 12 sessions, 1 h per session, over 4 weeks-CG: 12 sessions, 40–45 min per session, over 4 weeks	None	None	None
Ariza-Mateos et al., 2019	Spain	– EG1: chronic pelvic pain (16), 0% – EG2: chronic pelvic pain (16), 0% – CG: chronic pelvic pain (17), 0%	Yes, inclusion criteria (TSK > 33 points to be included)	FABQ-PA	– EG1: graded exposure and manual therapy – EG2: manual therapy – CG: educational booklet about chronic pelvic pain	– EG1: 12 sessions, 45 min per session, over 6 weeks – EG2: 18 sessions, 45 min per session, over 6 weeks – CG: over 6 weeks	3 months post intervention	NCT03590236	Not reported
Doğan et al., 2019	Turkey	– EG: cLBP (28), NIA – CG: cLBP (27), NIA	No	TSK	– EG: fascial manipulation techniques and usual physiotherapy treatment (hot pack, microwave diathermy, interferential flow-vacuum application, and exercises) – CG: usual physiotherapy treatment (hot pack, microwave diathermy, interferential flow-vacuum application, and exercises)	– EG: 15 sessions, over 3 weeks (physiotherapy) and 5 sessions, 10 min per session, over 3 weeks (fascial manipulation) – CG: 15 sessions, over 3 weeks	None	None	Not reported
Cruz-Diaz et al., 2018	Spain	– EG: cLBP (32), 34% – CG: cLBP (30), 33%	No	TSK	– EG: Pilates exercises – CG: educational booklet about cLBP	– EG: 24 sessions, 25 min per session, over 12 weeks – CG: overs 12 weeks	None	NCT02371837	None
Tüzün et al., 2017	Cyprus	– EG: cLBP (18), 56% – CG: cLBP (16), 25%	No	TSK	– EG: dry needling and classic massage – CG: physiotherapy (hot-pack, TENS, ultrasound) and home exercises at home	– EG: 6 sessions, over 3 weeks – CG: NIA	None	None	Not reported
Yilmaz Yelvar et al., 2017	Turkey	– EG: cLBP (22), 55% – CG: cLBP (22), 18%	No	TSK	– EG: immersive motor imagery, physiotherapy (hot-pack, TENS, ultrasound, therapeutic exercises) and exercises at home – CG: physiotherapy (hot-pack, TENS, ultrasound, therapeutic exercises) and exercises at home	– EG:10 sessions, over 2 weeks – CG: 10 sessions, over 2 weeks	None	None	Not reported
Buyukturan et al., 2017	Turkey	– EG: cNP with CDH (25), NIA – CG: cNP with CDH (25), NIA	No	TSK	– EG: core stability training and cervical stability training – CG: cervical stability training	– EG: 24 sessions, over 4 weeks – CG: 24 sessions, over 4 weeks	None	None	None
Keane Lynda, 2017	United Kingdom	– EG1: cLBP (10), 20% – EG2: cLBP (10), 20% – CG: cLBP (10), 11%	No	TSK	– EG1: aquatic stretching exercises – EG2: stretching exercises – CG: no intervention	– EG1: 24 sessions, 30 min per session, over 12 weeks – EG2: 24 sessions, 30 min per session, over 12 weeks – CG: overs 12 weeks	None	None	None
Oksuz et al., 2014	Turkey	– EG: osteoporosis pain (20), 0% – CG: osteoporosis pain (20), 0%	No	TSK	– EG: Pilates exercises – CG: no intervention	– EG: 18 sessions, 3 times per week, over 6 weeks – CG: over 6 weeks	None	None	None
Barnhoorn et al., 2015	Netherlands	– EG: CRPS-1 (35), 17% – CG: CRPS-1 (21), 24%	No	TSK	– EG: PEPT while CRPS-1 medication is stopped – CG: pharmacological intervention and physiotherapy	– EG: 5 sessions, 40 min per session – CG: NIA	3, 6 and 9 months post intervention	NCT00817128	Non-profit
Vincent et al., 2014	United States	– EG1: cLBP (17), 29% – EG2: cLBP (18), 32% – CG: cLBP (14), 39%	No	TSK	– EG1: total body resistance exercise and educational recommendations – EG2: lumbar extension resistance exercise and educational recommendations – CG: educational recommendations	– EG1: 48 sessions, over 16 weeks** – EG2: 42 sessions, over 16 weeks** – CG: over 16 weeks**	None	NCT01250262	Non-profit
Lara-Palomo et al., 2013	Spain	– EG: cLBP (30), 55% – CG: cLBP (31), 63%	No	TSK	– EG: electro-massage – CG: superficial massage	– EG: 20 sessions, 30 min per session, over 10 weeks – CG: 20 sessions, 20 min per session, over 10 weeks	None	None	None
Castro-Sánchez et al., 2012	Spain	– EG: cLBP (30), 30% – CG: cLBP (29), 34%	No	TSK	– EG: 4 I-strips were placed at 25% tension overlapping in a star shape over the point of maximum pain – CG: 1 I-strip applied transversely immediately above the point of maximum pain	– EG: the tape strips were applied once and left on the patient’s back for 7 days – CG: the tape strip was applied once and left on the patient’s back for 7 days	1 month post intervention	ACTRN1261200 0402842	None
Gustavsson and Koch, 2006	Sweden	– EG: cNP (13), 0% – CG: cNP (16), 7%	No	TSK	EG: relaxation training, body awareness exercises, pain and stress management education CG: usual physiotherapy care	– EG: 7 sessions, 90 min per session, over 7 weeks – CG: over 7 weeks	13 weeks post intervention	None	Non-profit

^†^Number of participants included in the analysis after the intervention. *Information confirmed by the authors. **According to the registration of the protocol. CDH, cervical disc herniation; CG, control group; cKP, chronic knee pain; cLBP, chronic low back pain; cNP, chronic neck pain; cPFP, chronic patellofemoral pain; CRPS-1, complex regional pain syndrome type 1; EG, experimental group; FABQ-PA, fear-avoidance beliefs questionnaire-physical activity; NIA, no information available; NMES, neuromuscular electrical stimulation; PEPT, pain exposure physical therapy; PNE, pain neurosciences education; TENS, transcutaneous electrical nerve stimulation; TMD, temporomandibular disorders; TSK, tampa scale kinesiophobia; VAS, visual analog scale; VR, virtual reality.

### Experimental and control interventions for kinesiophobia

Nineteen studies had one experimental intervention and eight studies had two, for a total of 35 experimental interventions (Table 2). These interventions were compared to sham comparator (*n* = 2, 7%), active comparator (*n* = 19, 70%), or no intervention control groups (*n* = 6, 22%). Of the two studies that used shams, Castro-Sánchez et al. (2012) applied kinesiology taping at the site of maximum pain in the lumbar area for both groups, which differed depending on the number of I-strips used (four for the experimental group, one for the sham group). Reynolds et al. (2020) also used a sham by performing cervical manipulations on patients with temporomandibular disorders for both groups, which differed based on the presence of high-velocity low-amplitude thrust (with thrust for the experimental group, without thrust for the sham group). As active comparators, nineteen of the included studies used standard approaches to treat kinesiophobia in patients with chronic pain (Table 2). These standard approaches included physiotherapeutic (84%), educational (5%), and multidisciplinary multimodal (11%) interventions. Experimental approaches included physiotherapeutic (69%), educational (3%), emotional (6%), psychological (3%), and multidisciplinary multimodal (20%) interventions (Table 2).

**TABLE 2:** Description of experimental and control interventions among the included studies.

Category	Experimental interventions (*n* = 35)	No of study	Control interventions, active comparators (*n* = 19)	No of study
Physical	– Aquatic exercise	1	– Aerobic and Pilates training	1
	– Cervical manipulation with thrust	1	– Conventional balance training and exercises	1
	– Dry needling and classic massage	1	– Dry needling and intramuscular electrical stimulation	1
	– Electro-massage	1	– Dynamic isometric exercises	1
	– Fascial manipulation techniques and physiotherapy	1	– Isotonic and isometric exercises	1
	– Immersive motor imagery and physiotherapy	1	– Neck stabilization exercises	1
	– Kinesiology tapping	1	– Physical exercises and TENS	1
	– Kinesiology tapping, dry needling and intramuscular electrical stimulation	1	– Physiotherapy	2
	– Manual therapy	1	– Physiotherapy (hot pack, microwave diathermy, interferential flow-vacuum application, and exercises)	1
	– Motor imagery	1	– Physiotherapy (hot pack, ultrasound, TENS, exercises program)	1
	– Neuromuscular electrical stimulation and physiotherapy	1	– Physiotherapy (hot-pack, TENS, ultrasound) and home exercises	1
	– Physical exercises	4	– Physiotherapy (hot-pack, TENS, ultrasound, therapeutic exercises) and home exercises	1
	– Physical exercises and TENS	1	– Physiotherapy (hot-pack, ultrasound, TENS) and home exercises	1
	– Physical exercises with swiss ball home exercises	1	– Stability training	1
	– Physical exercises with virtual reality	1	– Superficial massage	1
	– Physical exercises with virtual reality and physiotherapy	1		
	– Pilates exercises	3		
	– Resistance exercises	2		
Educational	– Pain education	1	– Usual treatment and a single education session	1
Emotional	– Relaxation technique	2		
Behavioral	– Pain exposure	1		
Multidisciplinary	– Pain education, pain exposure	1	– Medical consultations and pharmacological treatment	1
	– Relaxation technique, body awareness exercises, pain and stress management education	1	– Pharmacological intervention and physiotherapy	1
	– Meditation and physical exercises	1		
	Graded pain exposure and manual therapy	1		
	– Relaxation technique and physical exercises	1		
	– Pain education and physiotherapy	2		

In the case of the control interventions, only active comparators (standard therapy) were considered.

### Chronic pain conditions

**FIGURE 3:**
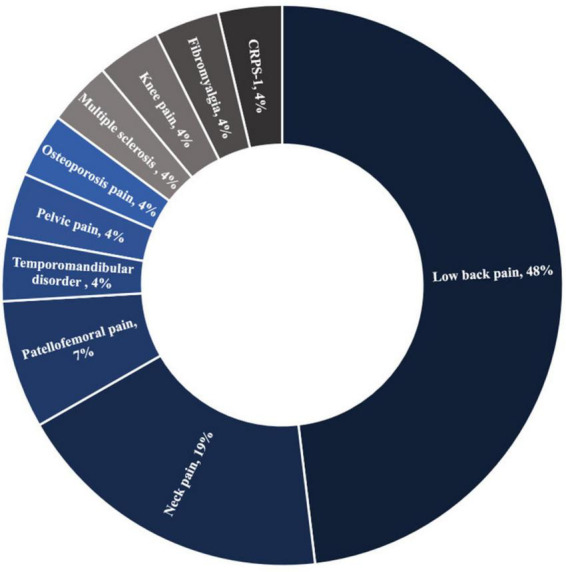
The relative distribution of chronic pain conditions in RCTs that were included (*n* = 27).

Half of the patients included in this review had chronic low back pain, and one-fifth had neck pain (Figure 3). Kinesiophobia was also targeted for other chronic musculoskeletal pain disorders such as patellofemoral pain, pelvic pain, osteoporosis pain, multiple sclerosis, knee pain, fibromyalgia, complex regional pain syndrome type 1, and temporomandibular disorder (Figure 3).

### Kinesiophobia assessment

**FIGURE 4:**
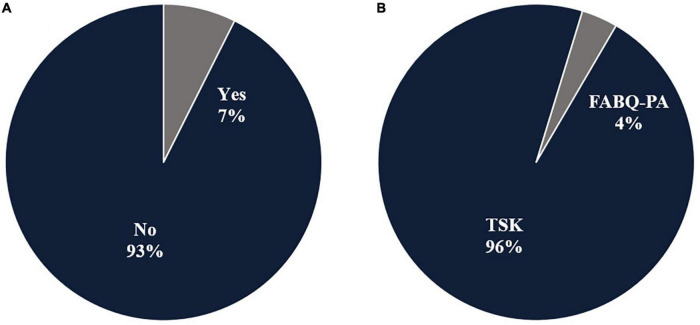
The relative distribution of included RCTs (*n* = 27) according to the following questions. **(A)** Was kinesiophobia a criterion for participants inclusion? **(B)** How is kinesiophobia measured? FABQ-PA, Fear avoidance beliefs questionnaire – physical activity scale; TSK, Tampa Scale of Kinesiophobia.

Two studies considered kinesiophobia as an eligibility criterion (Figure 4A). Participants in the study of Ariza-Mateos et al. (2019) had to have a Tampa Scale of Kinesiophobia (TSK) score greater than 33 points, while participants in the study of Moraes et al. (2021) had to have a TSK score greater than or equal to 51 points. One study used the Fear-Avoidance Beliefs Questionnaire’s physical activity subscale to assess the effect of the interventions on kinesiophobia, Ariza-Mateos et al. (2019), while the rest of studies used the TSK (Figure 4B).

## Discussion

### Overview of included studies

The purpose of this scoping review was to map out the literature on therapies for kinesiophobia in patients suffering from chronic pain, as well as to identify gaps in the literature and potential directions for future investigations. Twenty-seven peers reviewed RCTs were included with a total of 1,382 chronic musculoskeletal pain patients. Thirty-five experimental interventions were compared to 27 control interventions. The initial research questions are discussed in the following paragraphs, as well as intriguing findings from the analysis.

### Experimental and control interventions for kinesiophobia

This review’s first research question was: What types of interventions have been or are currently being studied in RCTs for the management of kinesiophobia in patients with chronic pain? Our results show that exposition to physical exercises is the most used approach to dealing with irrational fear of movement. However, given that pain and kinesiophobia are phenomena having a multifactorial origin with a significant role being played by biological, psychological, and social factors (Gatchel et al., 2007; Knapik et al., 2011), “one size does not fit all” when it comes to its management. Multidisciplinary therapies have received little attention in the reviewed studies, which have mostly focused on one type of intervention at a time. However, interesting and promising multidisciplinary designs stand out. For example, Moraes et al. (2021), collaborated with nurses treating chronic low back pain patients to develop a cognitive-behavioral therapy that combines pain education, pain exposure, and standard treatment (medical consultation and pharmacological treatment). Another study by Reynolds et al. (2020) looked at the efficacy of a combination of cervical thrust manipulation, behavioral education, soft tissue mobilization, and home exercises in the treatment of temporomandibular disorder. Gustavsson and Koch (2006) provided another example with their intervention in chronic neck pain patients that combined relaxation training, body awareness exercises, and pain and stress management education.

As the use of therapeutic interventions for kinesiophobia and chronic pain grows, guidelines for their development and evaluation must be established. Every biomedical experimental intervention should go through five phases, according to the U.S. Food and Drug Administration and the National Institute of Health (U.S. Food and Drug Administration, 2006, 2018; National Institutes of Health - National Institute on Aging., 2020). Phase 0 studies use a small sample (less than 15 patients) to formulate relevant hypotheses for further research. Phase I studies evaluate a new intervention’s feasibility and initial clinical efficacy in a small group of patients (20–100). Phase II studies aim to assess a new treatment’s efficacy in a larger group of people (from 100 to 300). Phase III studies evaluate a new treatment’s efficacy in large groups of people (300–3,000) while also monitoring side effects. Finally, Phase IV trials follow thousands of volunteers for years to assess safety and long-term effects.

Non-pharmacological investigations rarely reach Phase III, most likely due to technical, human, and financial challenges associated with these types of trials. All stages of the development of a new therapeutic intervention should include direct input from patients and end-users. Failures of new interventions can be partly explained by a non-adaptation to patients’ and users’ feedback (Birckhead et al., 2019). Incorporating patients and end-users into a co-construction design process can enable researchers to increase the relevance and effectiveness of their therapy (Birckhead et al., 2019).

### Chronic pain conditions

This review’s second research question was: What chronic pain conditions are targeted by these interventions? According to our findings most scientific efforts to treat kinesiophobia have thus far focused on musculoskeletal pain conditions, particularly low back pain and neck pain, which is consistent with the results of reviews by Watson et al. (2019); Hanel et al. (2020), and Kamonseki et al. (2021). These two conditions are widespread worldwide (Vos et al., 2012; Hoy et al., 2014), and account for 70% of all years lived with disability due to musculoskeletal disorders (Vos et al., 2012), which may explain why they have been the focus of extensive research. Despite their importance, other chronic pain disorders recently associated with kinesiophobia, such as cancer pain (Van der Gucht et al., 2020), neuropathic pain (Koca et al., 2019; Herrero-Montes et al., 2022), cephalalgia and orofacial pain (Kocjan, 2017; Benatto et al., 2019; Lira et al., 2019), would deserve more research interest.

### Kinesiophobia as an eligibility criterion

Even if kinesiophobia was considered as a primary outcome in all included studies, only two of them considered kinesiophobia as an eligibility criterion for participants’ selection. This presents a challenge when evaluating an intervention for kinesiophobia in participants who may or may not be kinesiophobia and brings us to the point where we must emphasize how important it is for investigators to define appropriate inclusion and exclusion criteria when designing a study (Patino and Ferreira, 2018). Inclusion criteria are the key characteristics of the target population that the researchers will use to answer their research question (Patino and Ferreira, 2018). The selection of the most appropriate inclusion/exclusion criteria should follow the process of identifying the selected primary outcome measure(s) (Jones et al., 2020). This approach of selecting the inclusion/exclusion criteria based on the primary outcome measure(s) reflects the importance of ensuring that research addresses the needs and concerns of those living with condition studied (Jones et al., 2020).

### Kinesiophobia assessment tools

This review’s third research question was: What assessment tools for kinesiophobia are used in these interventions? We found that the Tampa Scale of Kinesiophobia (TSK) was the most commonly used tool to assess kinesiophobia in the reviewed studies, which is consistent with previous findings (Tegner et al., 2018; Watson et al., 2019; Martinez-Calderon et al., 2020; Kamonseki et al., 2021). Other questionnaires, such as the Kinesiophobia Causes Scale (KCS) (Knapik et al., 2011) and the NeckPix (Monticone et al., 2015), could also be used for kinesiophobia assessment. Furthermore, the Fear-Avoidance of Pain Scale (FAPS) (Crowley and Kendall, 1999), the Fear of Pain Questionnaire (FPQ) (Tella et al., 2019), the Fear-Avoidance Beliefs Questionnaire (FABQ) (Waddell et al., 1993), and the Athlete Fear-Avoidance Questionnaire (AFAQ) (Dover and Amar, 2015) are all tools with a kinesiophobia subscale. For a comprehensive comparison of these instruments, we refer the reader to the article of Liu et al. (2021). Among the included studies, only one team, Ariza-Mateos et al. (2019), used one of these tools, the FABQ with the physical activity subscale.

These different questionnaires do not necessarily have the same underlying conceptual model (Lundberg et al., 2009, 2011), which makes their psychometric properties difficult to compare. Although the TSK-17 (17 questions) is the most popular, there are some drawbacks that patients and clinicians frequently report, such as a long completion time or a lack of sensitivity (Pincus et al., 2010; Wuttke, 2021). To address these concerns, the TSK-17 has been converted into several short versions. In the TSK-11, psychometrically poor items 4, 8, 9, 12, 14, and 16 were removed (Woby et al., 2005). These items demonstrated a low correlation between the question score and the overall assessment score, and/or response trends that deviated from a normal distribution pattern (Woby et al., 2005).

Given that kinesiophobia appears to be more than a simple fear of movement, but rather the expression of a complex and multifactorial mindset stemming from the belief of fragility and susceptibility to injury (Kori et al., 1990), it seems appropriate to consider and assess this clinical measure with a tool that can address the multifactorial aspects that comprise the kinesiophobia mindset. Since 2016, a new questionnaire called the Fear-Avoidance Components Scale (FACS) is beginning to be used and seems to be the most adequate tool to date to assess multi components of fear of movement mindset, with the most comprehensive scale and good psychometric characteristics (Neblett et al., 2016; Knezevic et al., 2018; Cuesta-Vargas et al., 2020). Despite limitations in the construct and empirical supports of kinesiophobia and, more broadly, fear-avoidance, all of these tools tend to assess and characterize a mindset that is clearly predictive disability over time (Crombez et al., 2012; Wideman et al., 2013). This highlights the importance to choose the best tool according to the study population and the research question.

### Interventions mainly studied in women

Our findings indicate a difference in the number of women and men who participated in the studies reviewed, with women accounting for 70% of the total sample size [refer also (Watson et al., 2019; Hanel et al., 2020; Martinez-Calderon et al., 2020)]. This difference could be explained by decades of epidemiological studies, which have reported higher prevalence of chronic pain in women compared to men for many different pain conditions (Rasmussen et al., 1991; Wolfe et al., 1995; LeResche, 1997; Bouhassira et al., 2008; Fillingim et al., 2009; Etherton et al., 2014; Mathieu et al., 2020). Sex disparities in pain experience have also been well documented, with women reporting more severe pain, at a higher frequency and greater duration on average, compared to men (Unruh, 1996). The actual literature is not successful in producing a clear and consistent pattern to explain these sexual dimorphisms in human pain sensitivity, possibly due to the multiple biological, psychosocial, and social factors interacting together to influence ascending and descending pain mechanisms (Popescu et al., 2010; Racine et al., 2012; Bartley and Fillingim, 2013).

### Limits

This review was limited to RCT. Due to publication bias; our review may also be unrepresentative of all completed studies. Indeed, our search strategy yielded a number of preliminary works presented in abstracts and clinical trial protocols, the results of which have not yet been published in peer-reviewed scientific journals (38% of excluded references). Moreover, an important difference among experimental and control interventions across studies is also important to consider in this review. Such issue stem in part from the fact that several research teams cannot afford iterative research development, challenging methodological consistency and replication.

### Recommendations for future studies

The relatively small number of RCTs identified regarding the broad field of kinesiophobia in adults with chronic pain highlights the importance of conducting future studies in this area. This relatively new field would benefit from replication and standardization as part of a theoretical framework to enable reflective and purposeful progress. A consensus on the best co-constructive research method for developing and evaluating new interventions for kinesiophobia within a scientific framework is required as guidelines developed for pharmacological studies are not the best suited for non-pharmacological trials. New RCTs evaluating person-centered, multidisciplinary intervention that takes into consideration the patient’s biological, psychological, and social experiences with pain and kinesiophobia are also required.

The different kinesiophobia assessment tools should be considered when designing a study, and the combination of several questionnaires should be considered, when necessary (Liu et al., 2021). Future studies should recruit a similar number of men and women to determine the effect of biological sex on the kinesiophobia intervention. Special attention should also be given to the various pathologies associated with chronic pain and kinesiophobia, other than chronic low back pain and chronic neck pain. Finally, authors of future studies should report their trial findings following standardized guidelines statements, such as the Consolidated Standards of Reporting Trials (CONSORT) for RCTs (Schulz et al., 2010) to facilitate the replicability of studies and the advancement of knowledge in the field.

## Conclusion

According to this scoping review of RCTs, the exposition to physical exercises is the most used approach to dealing with irrational fear of movement, and the Tampa Scale of Kinesiophobia is the most used tool to measure kinesiophobia. Management of kinesiophobia has so far largely focused on patients with musculoskeletal pain, particularly low back pain and neck pain. Future RCTs should consider the level of kinesiophobia as an eligibility criterion, as well as multidisciplinary interventions that can help patients confront their irrational fear of movement while considering the patient’s personal biological, psychological, and social experiences with pain and kinesiophobia.

## Author contributions

MB drafted the data collection tools, performed the literature search, participated and oversaw the data collection, analyzed the data, and wrote the first draft of the manuscript. AD, MV, SL, LS, and TB worked together to classify the references and to the data charting. MV assisted with data analysis. MV and AD assisted with the first draft of the manuscript. All authors contributed to the study’s design, development of the data collection tool, and manuscript revision and agreed on the final version of the submitted manuscript.

## Footnotes

1
https://osf.io/ktj84

## References

[B1] Akodu A. K. Nwanne C. A. Fapojuwo O. A. (2021). Efficacy of neck stabilization and Pilates exercises on pain, sleep disturbance and kinesiophobia in patients with non-specific chronic neck pain: A randomized controlled trial. *J. Bodyw. Mov. Ther.* 26 411–419. 10.1016/j.jbmt.2020.09.008 33992276

[B2] Ariza-Mateos M. J. Cabrera-Martos I. Ortiz-Rubio A. Torres-Sánchez I. Rodríguez-Torres J. Valenza M. C. (2019). Effects of a patient-centered graded exposure intervention added to manual therapy for women with chronic pelvic pain: A randomized controlled trial. *Arch. Phys. Med. Rehabil.* 100 9–16. 10.1016/j.apmr.2018.08.188 30312595

[B3] Aydoğan Arslan S. Demirgüç A. Kocaman A. Keskin E. (2020). The effect of short-term neuromuscular electrical stimulation on pain, physical performance, kinesiophobia, and quality of life in patients with knee osteoarthritis. *Physiotherapyq* 28 31–37. 10.5114/pq.2020.92477

[B4] Bagheri S. Naderi A. Mirali S. Calmeiro L. Brewer B. W. (2021). Adding mindfulness practice to exercise therapy for female recreational runners with patellofemoral pain: A randomized controlled trial. *J. Athl. Train.* 56 902–911. 10.4085/1062-6050-0214.20 33237990 PMC8359715

[B5] Barnhoorn K. J. Staal J. B. van Dongen R. T. Frölke J. P. Klomp F. P. van de Meent H. (2015). Are pain-related fears mediators for reducing disability and pain in patients with complex regional pain syndrome type 1? An explorative analysis on pain exposure physical therapy. *PLoS One* 10:e0123008. 10.1371/journal.pone.0123008 25919011 PMC4412526

[B6] Bartley E. J. Fillingim R. B. (2013). Sex differences in pain: A brief review of clinical and experimental findings. *Br. J. Anaesth.* 111 52–58. 10.1093/bja/aet127 23794645 PMC3690315

[B7] Benatto M. T. Bevilaqua-Grossi D. Carvalho G. F. Bragatto M. M. Pinheiro C. F. Straceri Lodovichi S. (2019). Kinesiophobia Is Associated with Migraine. *Pain Med.* 20 846–851. 10.1093/pm/pny206 30462312

[B8] Birckhead B. Khalil C. Liu X. Conovitz S. Rizzo A. Danovitch I. (2019). Recommendations for methodology of virtual reality clinical trials in health care by an international working group: Iterative study. *JMIR Ment. Health* 6:e11973. 10.2196/11973 30702436 PMC6374734

[B9] Bouhassira D. Lanteri-Minet M. Attal N. Laurent B. Touboul C. (2008). Prevalence of chronic pain with neuropathic characteristics in the general population. *Pain* 136 380–387. 10.1016/j.pain.2007.08.013 17888574

[B10] Bränström H. Fahlström M. (2008). Kinesiophobia in patients with chronic musculoskeletal pain: Differences between men and women. *J. Rehabil. Med.* 40 375–380. 10.2340/16501977-0186 18461263

[B11] Buyukturan B. Guclu - Gunduz A. Buyukturan O. Dadali Y. Bilgin S. Kurt E. E. (2017). Cervical stability training with and without core stability training for patients with cervical disc herniation: A randomized, single-blind study. *Eur. J. Pain* 21 1678–1687. 10.1002/ejp.1073 28730680

[B12] Castro-Sánchez A. M. Lara-Palomo I. C. Matarán- Peñarrocha G. A. Fernández-Sánchez M. Sánchez-Labraca N. Arroyo-Morales M. (2012). Kinesio taping reduces disability and pain slightly in chronic non-specific low back pain: A randomised trial. *J. Physiother.* 58 89–95. 10.1016/S1836-9553(12)70088-7 22613238

[B13] Crombez G. Eccleston C. Van Damme S. Vlaeyen J. W. S. Karoly P. (2012). Fear-avoidance model of chronic pain: The next generation. *Clin. J. Pain* 28 475–483. 10.1097/AJP.0b013e3182385392 22673479

[B14] Crowley D. Kendall N. A. S. (1999). Development and initial validation of a questionnaire for measuring fear-avoidance associated with pain: The fear-avoidance of pain scale. *J Musculoskelet. Pain* 7 3–19. 10.1300/J094v07n03_02

[B15] Cruz-Diaz D. Romeu M. Velasco-Gonzalez C. Martinez-Amat A. Hita-Contreras F. (2018). The effectiveness of 12 weeks of Pilates intervention on disability, pain and kinesiophobia in patients with chronic low back pain: A randomized controlled trial [with consumer summary]. *Clin. Rehabil.* 32 1249–1257. 10.1177/0269215518768393 29651872

[B16] Cuesta-Vargas A. I. Neblett R. Gatchel R. J. Roldán-Jiménez C. (2020). Cross-cultural adaptation and validity of the Spanish fear-avoidance components scale and clinical implications in primary care. *BMC Fam. Pract.* 21:44. 10.1186/s12875-020-01116-x 32106823 PMC7047382

[B17] Desrosiers M. (2018). *KINAP Évaluation de la kinésiophobie; Guide de l’intervenant.* Québec: Centre intégré universitaire de santé et de services sociaux (CIUSSS) de la Capitale-Nationale.

[B18] Doğan B. E. Bayramlar K. Turhan B. (2019). Investigation of fascial treatment effectiveness on pain, flexibility, functional level, and kinesiophobia in patients with chronic low back pain. *Physiotherapyq* 27:1. 10.5114/pq.2019.86461

[B19] Domingues de Freitas C. Costa D. A. Junior N. C. Civile V. T. (2020). Effects of the pilates method on kinesiophobia associated with chronic non-specific low back pain: Systematic review and meta-analysis. *J. Bodyw. Mov. Ther.* 24 300–306. 10.1016/j.jbmt.2020.05.005 32826004

[B20] Dover G. Amar V. (2015). Development and validation of the athlete fear avoidance questionnaire. *J. Athl. Train. (Allen Press)* 50 634–642. 10.4085/1062-6050-49.3.75 25793458 PMC4527448

[B21] Etherton J. Lawson M. Graham R. (2014). Individual and gender differences in subjective and objective indices of pain: Gender, fear of pain, pain catastrophizing and cardiovascular reactivity. *Appl. Psychophysiol. Biofeedback* 39 89–97. 10.1007/s10484-014-9245-x 24696322

[B22] Fillingim R. B. King C. D. Ribeiro-Dasilva M. C. Rahim-Williams B. Riley J. L. III (2009). Sex, gender, and pain: A review of recent clinical and experimental findings. *J. Pain* 10 447–485. 10.1016/j.jpain.2008.12.001 19411059 PMC2677686

[B23] Gatchel R. J. Peng Y. B. Peters M. L. Fuchs P. N. Turk D. C. (2007). The biopsychosocial approach to chronic pain: Scientific advances and future directions. *Psychol. Bull.* 133 581–624. 10.1037/0033-2909.133.4.581 17592957

[B24] Giusti E. M. Manna C. Varallo G. Cattivelli R. Manzoni G. M. Gabrielli S. (2020). The predictive role of executive functions and psychological factors on chronic pain after orthopaedic surgery: A longitudinal cohort study. *Brain Sci.* 10:685. 10.3390/brainsci10100685 32998214 PMC7601771

[B25] Gül H. Erel S. Toraman N. F. (2021). Physiotherapy combined with therapeutic neuroscience education versus physiotherapy alone for patients with chronic low back pain: A pilot, randomized-controlled trial. *Turk J. Phys. Med. Rehabil.* 67 283–290. 10.5606/tftrd.2021.5556 34870114 PMC8606998

[B26] Gulsen C. Soke F. Eldemir K. Apaydin Y. Ozkul C. Guclu-Gunduz A. (2020). Effect of fully immersive virtual reality treatment combined with exercise in fibromyalgia patients: A randomized controlled trial. *Assist. Technol.* 34, 256–263. 10.1080/10400435.2020.1772900 32543290

[B27] Gustavsson C. Koch Lv (2006). Applied relaxation in the treatment of long-lasting neck pain: A randomized controlled pilot study. *J. Rehabil. Med.* 38 100–107. 10.1080/16501970510044025 16546766

[B28] Haddaway N. McGuinness L. Page M. Pritchard C. (2020). *PRISMA 2020, R package and ShinyApp for making PRISMA 2020 flow diagrams.* Available online at: https://www.eshackathon.org/software/PRISMA2020.html (accessed February 2022).

[B29] Hanel J. Owen P. J. Held S. Tagliaferri S. D. Miller C. T. Donath L. (2020). Effects of exercise training on fear-avoidance in pain and pain-free populations: Systematic review and meta-analysis. *Sports Med.* 50 2193–2207. 10.1007/s40279-020-01345-1 32946074

[B30] Herrero-Montes M. Fernández-de-las-Peñas C. Ferrer-Pargada D. Tello-Mena S. Cancela-Cilleruelo I. Rodríguez-Jiménez J. (2022). Prevalence of neuropathic component in post-COVID pain symptoms in previously hospitalized COVID-19 survivors. *Int. J. Clin. Pract.* 2022:3532917. 10.1155/2022/3532917 35685491 PMC9159239

[B31] Hoy D. March L. Brooks P. Blystartrth F. Woolf A. Bain C. (2014). The global burden of low back pain: Estimates from the Global Burden of Disease 2010 study. *Ann. Rheum. Dis.* 73 968–974. 10.1136/annrheumdis-2013-204428 24665116

[B32] James J. Selfe J. Goodwin P. (2021). Does a bespoke education session change levels of catastrophizing, kinesiophobia and pain beliefs in patients with patellofemoral pain? A feasibility study. *Physiother. Pract. Res.* 42 153–163. 10.3233/PPR-210529

[B33] Javdaneh N. Molayei F. Kamranifraz N. (2021). Effect of adding motor imagery training to neck stabilization exercises on pain, disability and kinesiophobia in patients with chronic neck pain [with consumer summary]. *Complement. Ther. Clin. Pract.* 42:101263. 10.1016/j.ctcp.2020.101263 33276225

[B34] Jones A. P. Clayton D. Nkhoma G. Sherratt F. C. Peak M. Stones S. R. (2020). “Choosing a patient-important primary outcome measure,” in *Different corticosteroid induction regimens in children and young people with juvenile idiopathic arthritis: The SIRJIA mixed-methods feasibility study* (Southampton: NIHR Journals Library). 10.3310/hta24360 PMC7443738 32758350

[B35] Kamonseki D. H. Christenson P. Rezvanifar S. C. Calixtre L. B. (2021). Effects of manual therapy on fear avoidance, kinesiophobia and pain catastrophizing in individuals with chronic musculoskeletal pain: Systematic review and meta-analysis [with consumer summary]. *Musculoskelet. Sci. Pract.* 51 102311. 10.1016/j.msksp.2020.102311 33302214

[B36] Keane Lynda G. (2017). Comparing aquastretch with supervised land based stretching for chronic lower back pain. *J. Bodyw. Mov. Ther.* 21 297–305. 10.1016/j.jbmt.2016.07.004 28532872

[B37] Kesik G. Ozdemir L. Mungan Ozturk S. (2022). The effects of relaxation techniques on pain, fatigue, and kinesiophobia in multiple sclerosis patients: A 3-arm randomized trial. *J. Neurosci. Nurs.* 54 86–91. 10.1097/JNN.0000000000000620 35149625

[B38] Knapik A. Saulicz E. Gnat R. (2011). Kinesiophobia – introducing a new diagnostic tool. *J. Hum. Kinet.* 28 25–31. 10.2478/v10078-011-0019-8 23487514 PMC3592098

[B39] Knezevic A. Neblett R. Gatchel R. J. Jeremic-Knezevic M. Bugarski-Ignjatovic V. Tomasevic-Todorovic S. (2018). Psychometric validation of the Serbian version of the Fear Avoidance Component Scale (FACS). *PLoS One* 13:e0204311. 10.1371/journal.pone.0204311 30248127 PMC6152979

[B40] Koca T. T. GÜLkesen A. NacİTarhan V. Koca Ö (2019). Does kinesiophobia associated with poststroke neuropathic pain and stroke severity? *J. Phys. Med. Rehabil. Sci.* 22 60–65. 10.31609/jpmrs.2019-66862

[B41] Kocjan J. (2017). Kinesiophobia (fear of movement) level among patients with diagnosis of cervicogenic headache. *J. Educ. Health Sport* 7 390–397.

[B42] Kori S. H. Miller R. P. Todd D. D. (1990). Kinisophobia: A new view of chronic pain behavior. *Pain Manag.* 3 35–43.

[B43] Lara-Palomo I. C. Aguilar-Ferrandiz M. E. Mataran-Penarrocha G. A. Saavedra-Hernandez M. Granero-Molina J. Fernandez-Sola C. (2013). Short-term effects of interferential current electro-massage in adults with chronic non-specific low back pain: A randomized controlled trial [with consumer summary]. *Clin. Rehabil.* 27 439–449. 10.1177/0269215512460780 23035006

[B44] Lara-Palomo I. C. Antequera-Soler E. Matarán-Peñarrocha G. A. Fernández-Sánchez M. García-López H. Castro-Sánchez A. M. (2022). Comparison of the effectiveness of an e-health program versus a home rehabilitation program in patients with chronic low back pain: A double blind randomized controlled trial. *Digit Health* 8:20552076221074482. 10.1177/20552076221074482 35111332 PMC8801654

[B45] Larsson C. Hansson E. E. Sundquist K. Jakobsson U. Ekvall Hansson E. (2016). Kinesiophobia and its relation to pain characteristics and cognitive affective variables in older adults with chronic pain. *BMC Geriatr.* 16 1–7. 10.1186/s12877-016-0302-6 27387557 PMC4936054

[B46] LeResche L. (1997). Epidemiology of temporomandibular disorders: Implications for the investigation of etiologic factors. *Crit. Rev. Oral Biol. Med.* 8 291–305. 10.1177/10454411970080030401 9260045

[B47] Lethem J. Slade P. D. Troup J. D. Bentley G. (1983). Outline of a fear-avoidance model of exaggerated pain perception–I. *Behav. Res. Ther.* 21 401–408. 10.1016/0005-7967(83)90009-8 6626110

[B48] Linton S. J. Shaw W. S. (2011). Impact of psychological factors in the experience of pain. *Phys. Ther.* 91 700–711. 10.2522/ptj.20100330 21451097

[B49] Lira M. R. Lemes da Silva R. R. Bataglion C. Aguiar A. D. S. Greghi S. M. Chaves T. C. (2019). Multiple diagnoses, increased kinesiophobia? - Patients with high kinesiophobia levels showed a greater number of temporomandibular disorder diagnoses. *Musculoskelet. Sci. Pract.* 44:102054. 10.1016/j.msksp.2019.102054 31491618

[B50] Liu H. Huang L. Yang Z. Li H. Wang Z. Peng L. (2021). Fear of movement/(Re)injury: An update to descriptive review of the related measures. *Front. Psychol.* 12 696762. 10.3389/fpsyg.2021.696762 34305755 PMC8292789

[B51] Lundberg M. K. E. Larsson M. Östlund H. Styf J. (2006). Kinesiophobia among patients with musculoskeletal pain in primary healthcare. *J. Rehabil. Med.* 38 37–43. 10.1080/16501970510041253 16548085

[B52] Lundberg M. Grimby-Ekman A. Verbunt J. Simmonds M. J. (2011). Pain-related fear: A critical review of the related measures. *Pain Res. Treat.* 2011:494196. 10.1155/2011/494196 22191022 PMC3236324

[B53] Lundberg M. Styf J. Jansson B. (2009). On what patients does the Tampa Scale for Kinesiophobia fit? *Physiother. Theory Pract.* 25 495–506. 10.3109/09593980802662160 19925172

[B54] Martinez-Calderon J. Flores-Cortes M. Morales-Asencio J. M. Luque-Suarez A. (2020). Conservative interventions reduce fear in individuals with chronic low back pain: A systematic review. *Arch. Phys. Med. Rehabil.* 101 329–358. 10.1016/j.apmr.2019.08.470 31473206

[B55] Mathieu S. Couderc M. Pereira B. Dubost J. J. Malochet-Guinamand S. Tournadre A. (2020). Prevalence of migraine and neuropathic pain in rheumatic diseases. *J. Clin. Med.* 9:1890. 10.3390/jcm9061890 32560321 PMC7356241

[B57] McGowan J. Sampson M. Salzwedel D. M. Cogo E. Foerster V. Lefebvre C. (2016). PRESS peer review of electronic search strategies: 2015 guideline statement. *J. Clin. Epidemiol.* 75 40–46. 10.1016/j.jclinepi.2016.01.021 27005575

[B58] Monticone M. Vernon H. Brunati R. Rocca B. Ferrante S. (2015). The NeckPix(©): Development of an evaluation tool for assessing kinesiophobia in subjects with chronic neck pain. *Eur. Spine J.* 24 72–79. 10.1007/s00586-014-3509-2 25115918

[B59] Moraes ÉB. Martins Junior F. F. Silva L. B. D. Garcia J. B. S. Mattos-Pimenta C. A. (2021). Self-efficacy and fear of pain to movement in chronic low back pain: An intervention developed by nurses. *Rev. Gaucha Enferm.* 42:e20200180. 10.1590/1983-1447.2021.20200180 34878010

[B60] Nambi G. Abdelbasset W. K. Alsubaie S. F. Saleh A. K. Verma A. Abdelaziz M. A. (2021). Short-term psychological and hormonal effects of virtual reality training on chronic low back pain in soccer players. *J. Sport Rehabil.* 30 884–893. 10.1123/jsr.2020-0075 33596538

[B61] National Institutes of Health - National Institute on Aging. (2020). *What are clinical trials and studies?.* Available Onlline at: https://www.nia.nih.gov/health/what-are-clinical-trials-and-studies (accessed April 4, 2022).

[B62] Neblett R. Mayer T. G. Hartzell M. M. Williams M. J. Gatchel R. J. (2016). The Fear-avoidance Components Scale (FACS): Development and psychometric evaluation of a new measure of pain-related fear avoidance. *Pain Pract.* 16 435–450. 10.1111/papr.12333 26228238

[B63] Oksuz S. Unal E. Dizmek P. Aydin Ozcan D. (2014). The effects of clinical pilates exercises on kinesophobia in women with osteoporosis. *Ann. Rheum. Dis.* 26:68e72. 10.1136/annrheumdis-2014-eular.4796

[B64] Özer D. Toprak Çelenay Ç. (2019). Effectiveness of relaxation training in addition to stabilization exercises in chronic neck pain: A randomized clinical trial. *Türk Fizyoterapi Rehabil. Dergisi.* 30 145–153. 10.21653/tjpr.665131

[B66] Patino C. M. Ferreira J. C. (2018). Inclusion and exclusion criteria in research studies: Definitions and why they matter. *J. Bras. Pneumol.* 44:84. 10.1590/s1806-37562018000000088 29791550 PMC6044655

[B67] Perrot S. Trouvin A.-P. Rondeau V. Chartier I. Arnaud R. Milon J.-Y. (2018). Kinesiophobia and physical therapy-related pain in musculoskeletal pain: A national multicenter cohort study on patients and their general physicians. *Joint Bone Spine* 85 101–107. 10.1016/j.jbspin.2016.12.014 28062380

[B68] Peters M. D. Godfrey C. McInerney P. Baldini Soares C. Khalil H. Parker D. (2017). “Scoping Reviews (2020 version), “in *Scoping Reviews (2020 version)*, eds Aromataris E. Munn Z. (Adelaide: JBI).

[B69] Pincus T. Smeets R. J. E. M. Simmonds M. J. Sullivan M. J. L. (2010). The fear avoidance model disentangled: Improving the clinical utility of the fear avoidance model. *Clin. J. Pain* 26 739–746. 10.1097/AJP.0b013e3181f15d45 20842017

[B70] Popescu A. LeResche L. Truelove E. L. Drangsholt M. T. (2010). Gender differences in pain modulation by diffuse noxious inhibitory controls: A systematic review. *Pain* 150 309–318. 10.1016/j.pain.2010.05.013 20557999

[B71] Racine M. Tousignant-Laflamme Y. Kloda L. A. Dion D. Dupuis G. Choiniere M. A. (2012). systematic literature review of 10 years of research on sex/gender and pain perception - part 2: Do biopsychosocial factors alter pain sensitivity differently in women and men? *Pain* 153 619–635. 10.1016/j.pain.2011.11.026 22236999

[B72] Rasmussen B. K. Jensen R. Schroll M. Olesen J. (1991). Epidemiology of headache in a general population–a prevalence study. *J. Clin. Epidemiol.* 44 1147–1157. 10.1016/0895-4356(91)90147-2 1941010

[B73] Reynolds B. Puentedura E. J. Kolber M. J. Cleland J. A. (2020). Effectiveness of cervical spine high velocity low amplitude thrust added to behavioral education, soft tissue mobilization, and exercise in individuals with temporomandibular disorder (TMD) with myalgia: A randomized clinical trial. *J. Orthop. Sports Phys. Ther.* 50 455–465. 10.2519/jospt.2020.9175 31905097

[B74] Schulz K. F. Altman D. G. Moher D. (2010). CONSORT 2010 Statement: Updated guidelines for reporting parallel group randomised trials. *BMC Med.* 8:18. 10.1186/1741-7015-8-18 20334633 PMC2860339

[B75] Tegner H. Frederiksen P. Esbensen B. A. Juhl C. (2018). Neurophysiological pain education for patients with chronic low back pain: A systematic review and meta-analysis. *Clin. J. Pain* 34 778–786. 10.1097/AJP.0000000000000594 29443723

[B76] Tella M. D. Ghiggia A. Testa S. Castelli L. Adenzato M. (2019). The fear of pain questionnaire: Factor structure, validity and reliability of the Italian translation. *PLoS One* 14:e0210757. 10.1371/journal.pone.0210757 30682182 PMC6347221

[B77] Thomson Reuters (2019). *Endnote, version X9.* Available online at: https://endnote.com/downloads

[B78] Tricco A. C. Lillie E. Zarin W. O’Brien K. K. Colquhoun H. Levac D. (2018). PRISMA extension for scoping reviews (PRISMA-ScR): Checklist and explanation. *Ann. Intern. Med.* 169 467–473. 10.7326/M18-0850 30178033

[B79] Trinderup J. S. Fisker A. Juhl C. B. Petersen T. (2018). Fear avoidance beliefs as a predictor for long-term sick leave, disability and pain in patients with chronic low back pain. *BMC Musculoskelet. Disord.* 19:431. 10.1186/s12891-018-2351-9 30509231 PMC6278039

[B80] Tüzün E. H. Gıldır S. Angin E. Tecer B. H. Dana K. Ö Malkoç M. (2017). Effectiveness of dry needling versus a classical physiotherapy program in patients with chronic low-back pain: A single-blind, randomized, controlled trial. *J. Phys. Ther. Sci.* 29 1502–1509. 10.1589/jpts.29.1502 28931976 PMC5599809

[B81] U.S. Food and Drug Administration (2006). *Exploratory IND studies. Guidance for industry, investigators, and reviewers.* Silver Spring, MD: FDA.

[B82] U.S. Food and Drug Administration (2018). *Step 3: Clinical Research.* Silver Spring, MD: FDA.

[B83] Unruh A. M. (1996). Gender variations in clinical pain experience. *Pain* 65 123–167. 10.1016/0304-3959(95)00214-6 8826503

[B84] Van der Gucht E. Dams L. Meeus M. Devoogdt N. Beintema A. Penen F. (2020). Kinesiophobia contributes to pain-related disability in breast cancer survivors: A cross-sectional study. *Support Care Cancer* 28 4501–4508. 10.1007/s00520-020-05304-4 31953624

[B85] Varallo G. Giusti E. M. Scarpina F. Cattivelli R. Capodaglio P. Castelnuovo G. (2020). The association of kinesiophobia and pain catastrophizing with pain-related disability and pain intensity in obesity and chronic lower-back pain. *Brain Sci.* 11:11. 10.3390/brainsci11010011 33374178 PMC7823580

[B86] Varallo G. Scarpina F. Giusti E. M. Suso-Ribera C. Cattivelli R. Guerrini Usubini A. (2021b). The role of pain catastrophizing and pain acceptance in performance-based and self-reported physical functioning in individuals with fibromyalgia and obesity. *J. Pers. Med.* 11:180. 10.3390/jpm11080810 34442454 PMC8401554

[B87] Varallo G. Scarpina F. Giusti E. M. Cattivelli R. Usubini A. G. Capodaglio P. (2021a). Does kinesiophobia mediate the relationship between pain intensity and disability in individuals with chronic low-back pain and obesity? *Brain Sci.* 11:684. 10.3390/brainsci11060684 34067433 PMC8224628

[B88] Vincent H. K. George S. Z. Seay A. N. Vincent K. R. Hurley R. W. (2014). Resistance exercise, disability, and pain catastrophizing in obese adults with back pain. *Med. Sci. Sports Exerc.* 46 1693–1701. 10.1249/MSS.0000000000000294 25133997 PMC4137474

[B89] Vlaeyen J. W. S. Kole-Snijders A. M. J. Rotteveel A. M. Ruesink R. Heuts P. H. T. G. (1995). The role of fear of movement/(re)injury in pain disability. *J. Occup. Rehabil.* 5 235–252. 10.1007/BF02109988 24234727

[B90] Vlaeyen J. W. Linton S. J. (2012). Fear-avoidance model of chronic musculoskeletal pain: 12 years on. *Pain* 153 1144–1147. 10.1016/j.pain.2011.12.009 22321917

[B91] Vos T. Flaxman A. D. Naghavi M. Lozano R. Michaud C. Ezzati M. (2012). Years lived with disability (YLDs) for 1160 sequelae of 289 diseases and injuries 1990-2010: A systematic analysis for the Global Burden of Disease Study 2010. *Lancet* 380 2163–2196. 10.1016/S0140-6736(12)61729-2 23245607 PMC6350784

[B92] Waddell G. Newton M. Henderson I. Somerville D. Main C. J. A. (1993). Fear-Avoidance Beliefs Questionnaire (FABQ) and the role of fear-avoidance beliefs in chronic low back pain and disability. *Pain* 52 157–168. 10.1016/0304-3959(93)90127-B 8455963

[B93] Watson J. A. Ryan C. G. Cooper L. Ellington D. Whittle R. Lavender M. (2019). Pain neuroscience education for adults with chronic musculoskeletal pain: A mixed-methods systematic review and meta-analysis [with consumer summary]. *J. Pain* 20 e1140e1–e1140e22. 10.1016/j.jpain.2019.02.011 30831273

[B94] Wideman T. H. Asmundson G. G. J. Smeets R. J. E. M. Zautra A. J. Simmonds M. J. Sullivan M. J. L. (2013). Re-thinking the fear avoidance model: Toward a multi-dimensional framework of pain-related disability. *Pain* 154 2262–2265. 10.1016/j.pain.2013.06.005 23748115 PMC4078976

[B95] Woby S. R. Roach N. K. Urmston M. Watson P. J. (2005). Psychometric properties of the TSK-11: A shortened version of the Tampa Scale for Kinesiophobia. *Pain* 117 137–144. 10.1016/j.pain.2005.05.029 16055269

[B96] Wolfe F. Ross K. Anderson J. Russell I. J. Hebert L. (1995). The prevalence and characteristics of fibromyalgia in the general population. *Arthritis Rheum.* 38 19–28. 10.1002/art.1780380104 7818567

[B97] Wuttke C. (2021). *Enquête sur l’utilité d’une échelle d’évaluation de la kinésiophobie : La TSK.* Nancy: Institut lorrain de formation en masso-kinésithérapie de Nancy.

[B98] Yilmaz Yelvar G. Çırak Y. Dalkılınç M. Parlak Demir Y. Guner Z. Boydak A. (2017). Is physiotherapy integrated virtual walking effective on pain, function, and kinesiophobia in patients with non-specific low-back pain? Randomised controlled trial. *Eur. Spine J.* 26 538–545. 10.1007/s00586-016-4892-7 27981455

