# Supplementary material for: Treatments for kinesiophobia in people with chronic pain: A scoping review
Source: Front Behav Neurosci. 2022 Sep 20;16:933483. doi: 10.3389/fnbeh.2022.933483 (PMC9531655; doi:10.3389/fnbeh.2022.933483)
Supplement: Supplementary file 1 [file Table_1.PDF]

Table S1. Example of full search strategy used for PubMed (Medline).

**Search**	**Query**	**Items found**
#1	("Pain"[Mesh:NoExp] OR "pain"[Title/Abstract] OR "pain"[Other Term] OR "ache*"[Title/Abstract] OR "ache*"[Other Term] OR "physical suffering*"[Title/Abstract] OR " physical suffering*"[Other Term] OR "myalgi*"[Title/Abstract] OR "myalgi*"[Other term] OR "Phantom Limb"[Mesh] OR "Musculoskeletal Pain"[Mesh] OR "Back Pain"[Mesh] OR "Cancer Pain"[Mesh] OR "Chronic Pain"[Mesh] OR "Pain, Intractable"[Mesh] OR "Complex Regional Pain Syndromes"[Mesh] OR "Causalgia"[Title/Abstract] OR "Causalgia"[Other Term] OR "Reflex Sympathetic Dystrophy"[Title/Abstract] OR "Reflex Sympathetic Dystrophy"[Other Term] OR "Neuralgia"[Mesh] OR "Neuralgia"[Title/Abstract] OR "Neuralgia"[Other Term] OR "algia*"[Title/Abstract] OR "algia*"[Other Term] OR "sciatica"[Title/Abstract] OR "sciatica"[Other Term] OR "Arthralgia"[Mesh] OR "Arthralgia"[Title/Abstract] OR "Arthralgia"[Other Term] OR "neuropath*"[Title/Abstract] OR "neuropath*"[Other Term] OR "Small Fiber Neuropathy"[Mesh] OR "Small Fiber Neuropath*"[Title/Abstract] OR "Small Fiber Neuropath*"[Other Term] OR "small nerve fiber neuropath*"[Title/Abstract] OR "Small nerve Fiber Neuropath*"[Other Term] OR "Arthritis"[Mesh:NoExp] OR "Arthritis, Rheumatoid"[Mesh] OR "Osteoarthritis"[Mesh] OR "Osteoarthritis"[Title/Abstract] OR "Osteoarthritis"[Other Term] OR "Periarthritis"[Mesh] OR "Periarthritis"[Title/Abstract] OR "Periarthritis"[Other Term] OR "Sacroiliitis"[Mesh] OR "Sacroiliitis"[Title/Abstract] OR "Sacroiliitis"[Other Term] OR "Spondylarthritis"[Mesh] OR "Spondylarthritis"[Title/Abstract] OR "Spondylarthritis"[Other Term] OR "arthriti*"[Title/Abstract] OR "arthriti*"[Other Term] OR "Polyarthritis"[Title/Abstract] OR "Polyarthritis"[Other Term] OR "failed back surgery syndrome"[Title/Abstract] OR "Failed Back Surgery Syndrome"[Other Term])	1,166,123
#2	("kinesiophobi*"[Title/Abstract] OR "kinesiophobi*"[Other term] OR "fear of movement"[Title/Abstract] OR "fear of movement"[Other term] OR "fear of fall*"[Title/Abstract] OR "fear of fall*"[Other term] OR "fear of reinjur*"[Title/Abstract] OR "fear of reinjur*"[Other term] OR ("fear"[MeSH Terms] AND "movement"[MeSH Terms]) OR ("fear"[Title/Abstract] AND "movement"[Title/Abstract]) OR ("fear"[Other term] AND "movement"[Other term]) OR ("fear"[Title] AND "reinjur*"[Title]) OR ("fear"[Title/Abstract] AND "reinjur*"[Title/Abstract]) OR ("fear"[Other term] AND "reinjur*"[Other term]) OR ("fear"[Title] AND "fall*"[Title]) OR ("fear"[Title/Abstract] AND "fall*"[Title/Abstract]) OR ("fear"[Other term] AND "fall*"[Other term]) OR "Tampa Scale"[Title/Abstract] OR "Tampa Scale"[Other term] OR "TSK"[Title/Abstract] OR "TSK"[Other term] OR "NeckPix"[Title/Abstract] OR "NeckPix"[Other term])	10,131
#3	("effect"[Title/Abstract] OR "trial"[Title/Abstract] OR "investigat*"[Title/Abstract] OR "random"[Title/Abstract] OR "control"[Title/Abstract] OR "experimental"[Title/Abstract] OR "double blind"[Title/Abstract] OR "compar"[Title/Abstract] OR "matched"[Title/Abstract] OR "blind"[Title/Abstract] OR "examine"[Title/Abstract] OR "study"[Title/Abstract] OR "comparative study"[Publication Type] OR "randomized controlled trial"[Publication Type])	14,815,796
#4	#1 AND #2 AND #3	1,858
